# The Need for a Competence Matrix in Delivery Rooms for the Proper Work of Multiprofessional Teams

**DOI:** 10.1055/s-0039-1677882

**Published:** 2019-01

**Authors:** Marcos Felipe Silva de Sá, Gustavo Salata Romão

**Affiliations:** 1 Faculdade de Medicina de Ribeirão Preto, Universidade de São Paulo, Ribeirão Preto, SP, Brazil; 2 Universidade de Ribeirão Preto, Ribeirão Preto, SP, Brazil

The Brazilian Ministry of Health (MH) is, although belatedly, actually concerned about the quality of maternal and childcare. Indeed, the Brazilian national indicators related to maternal and child mortality, the low quality of prenatal care, illustrated by the sharp growth in the number of cases of syphilis in pregnant women and congenital syphilis in recent years, the high rates of cesarean sections (C-sections; Brazil is the second country in C-section rates in the world), and the poor quality of delivery care, so broadly and repeatedly exposed in the media, are alarming. There is an endless number of failures that may be attributed to the poor performance of the Brazilian health system, including private health insurance plans.

Regarding delivery care, it is widely known that it requires more attention, taking into account the situation of imminent risk to which the mother and her fetus are exposed. The World Health Organization (WHO) has recently issued a publication entitled *Intrapartum Care for a Positive Childbirth Experience*,^1^ with recommendations for care during labor and delivery based on an extensive review of published studies, as well as care protocols from several countries. These data were submitted to evaluation by an external commission, which included experts from the International Federation of Gynecology and Obstetrics (known by its French acronym FIGO). The WHO document was analyzed and endorsed by the Brazilian Federation of Gynecology and Obstetrics Associations' (Febrasgo, in Portuguese) National Specialized Commission for Childbirth and Postpartum Care.^2^ It establishes the recommended and non-recommended practices according to the 2018 WHO intrapartum care model (considering healthy mothers and fetuses or newborns). Other articles about good practices in delivery care can be found in some publications in the Brazilian literature.^3^

In Brazil, governmental and nongovernmental organizations have proposed several actions to improve obstetric care and reduce C-section rates, with many programs currently in progress. In 2011, the MH launched the ‘Rede Cegonha’ program, which is developed in hospitals linked to the Brazilian Unified Health System (SUS, in Portuguese). The objective of this program is to ensure that all women have access to comprehensive health care since pregnancy confirmation up to the second year of life of the child. It implies the guarantee of access and improvement in the quality of childbirth care.^4^ Recently, the ‘Parto Cuidadoso’ program, whose objectives are humanizing care in public maternities, was linked to ‘Rede Cegonha,’ and, in addition to improving the care offered to pregnant and parturient women, its secondary goal is reducing C-section rates.

In 2015, the ‘Parto Adequado’ project, which was developed by the Brazilian National Regulatory Agency for Private Health Insurance and Plans (ANS, in Portuguese), Hospital Israelita Albert Einstein, and the Institute for Healthcare Improvement (US), was implemented, with the support of the MH, to identify innovative and feasible models of childbirth care that value normal childbirth and promote a decrease in the percentage of C-sections. In the first stage, 35 large hospitals and 19 health insurance operators were selected for the program. In the second phase, which is currently in progress, 137 private hospitals, 25 public hospitals, 65 health insurance operators, and 73 associated hospitals were involved in the program, with a significant reduction in the number of C-sections in the institutions in which it was applied.^5^

At the SUS setting, the ‘Aprimoramento e Inovação no Cuidado e Ensino em Obstetrícia e Neonatologia’ (Apice On, in Portuguese) project, an initiative of the MH in partnership with Empresa Brasileira de Serviços Hospitalares (EBSERH, in Portuguese), Associação Brasileira de Hospitais Universitários e de Ensino (ABRAHUE, in Portuguese), the Brazilian Ministry of Education (MEC, in Portuguese), and Instituto Fernandes Figueira/Fundação Oswaldo Cruz (IFF/FIOCRUZ, in Portuguese), was created in 2017, with Universidade Federal de Minas Gerais as the executing institution.^6^ According to the MH, ‘Apice On proposes training in the areas of childbirth care, postpartum and postmiscarriage reproductive planning, and care to women who have experienced sexual violence, miscarriage or legal abortion, in teaching or university hospitals, or in auxiliary teaching units, within the scope of “Rede Cegonha.” Its purposes are expanding the reach of the activities developed by hospitals from the SUS network and reformulating and/or improving work processes and flows to adjust care access, coverage, and quality.’

Consequently, with the objective to contribute to the programs designed to improve obstetric care in Brazil and reduce C-section rates, Febrasgo has actively supported the ‘Parto Adequado’ and Apice On projects. We understand that, when proposing or implementing public policies for maternal and childcare, public managers must seek to establish communications with professionals in the area, in a coordinated way, so they can all participate in the formulation of standards, ordinances, laws etc., engaging and encouraging them to play a more active role in maternal and childcare. Obstetricians access the Febrasgo portal on a regular basis, and periodically receive varied types of information: notices of conferences, scientific texts, protocols, newsletters, direct mailing, and journals. Therefore, Febrasgo intends to be a mediator partner in the actions of the MH in the women's health area, that is, a real bridge joining the public managers and the 16,000 gynecologist and obstetrician associate professionals who care for patients at clinics, wards, offices, and delivery rooms, public or not. They are the specialists that push the health system forward regarding women's health programs.

The Febrasgo, in consonance with the aforementioned projects, has repeatedly expressed its support to the work performed by multiprofessional teams, including obstetricians, nurse midwives, anesthetists, neonatologists, social workers and others, in delivery rooms. However, in order for this integrated work to be successful, the interaction of all of the participants in establishing a routine for the delivery room is necessary. Obstetricians are the only professionals trained to act throughout all of the childbirth process, from conception to postpartum. Therefore, there is no reason to fear conjoint work. On the contrary, interdisciplinary action allows obstetricians to work longer where their skills are essential. The patients certainly will notice the better care. This does not mean that physicians should refrain from the responsibility of following the childbirth process. By isolating themselves in the process, resisting to multiprofessional integration, obstetricians will keep losing their role in the group, compromising care quality and safety.

Non-medical professionals have been introduced in the process of providing care to pregnant women and parturients as a measure to ‘save the system.’ However, the insertion of new professionals should necessarily be accompanied by an adjustment, with specifications of the assignments of each professional involved in the care of pregnant women, parturients, and newborns, and with each professional doing their share in a coordinated way. If the group of professionals works in unison, better results are expected regarding the well-being of mothers and fetuses. By ‘forcing’ the insertion of non-medical professionals in delivery rooms without the previously-established support of an obstetrician, the lives of pregnant women and fetuses may be put at risk. The result of this hasty action is a sequence of judicial conflicts that involve regional or federal councils of non-medical professionals with the Brazilian Federal Medical Council, a staunch defender of the Medical Act Law, the norms that regulate medical practice in Brazil.^7^ These judicial conflicts become more frequent, especially when the complications occur during childbirths assisted by non-medical professionals and that demand the intervention of an obstetrician. Additionally, interpersonal conflicts often arise from the poor integration among different professionals.

Based on these facts and since many of these programs are developed in public hospitals and maternities, usually university institutions, in order to minimize these problems, we suggest that the managers should formulate the program with the participation of representatives from all of the professions involved, under the coordination of the clinical director of each hospital or maternity unit. Thus, a competence matrix for the delivery room should be developed for this purpose. The role of each professional in the activities developed in this setting would be well-defined since the arrival of the pregnant woman or parturient at the hospital, the labor, the childbirth, the reception of the newborn and the postpartum steps, up to hospital discharge. With this document, the hierarchical levels of competence of each professional would be respected, in accordance with the laws that regulate the involved professions. The work performed by an interdisciplinary group of professionals has greater chances of being successful and integrating the group better.

The Febrasgo has recently developed a competence matrix for training resident (R) physicians in gynecology and obstetrics, based on The Obstetrics and Gynecology Milestone Project, which was designed by the American Board of Obstetrics and Gynecology (ABOG) and the American College of Obstetrics and Gynecology (ACOG);^8^ it establishes the roles of R1, R2, and R3 in the service routine, by training level and technical qualification, and according to their knowledges, skills, and decision-making ability.^9^ The Febrasgo competence matrix contains an innovative axis entitled ‘Professionalism,’ whose objective is to strengthen new frameworks in global health, humanization, and care qualification, emphasizing teamwork, with professionals who complete medical residency programs in the gynecology and obstetrics field.

Based on that document, we suggested that the formulation of the competence matrix in the delivery room take into account some requisites in the evaluation of medical or non-medical professional capacity, which may be essential and should include key aspects such as: 1) understanding the roles of the care team members; 2) capacity to communicate effectively within the team; 3) understanding the importance of care duties (shift changes and referrals) and team meetings; 4) punctuality in the clinical activities; 5) proper and timely filling of administrative and medical records and reports; 6) effective performance in interprofessional and interdisciplinary health teams; 7) effective communication with physicians and other healthcare professionals regarding the care provided to patients; 8) engagement in shared decision-making, taking into consideration the cultural characteristics of the patients and their families; 9) organization of and participation in guidance actions in multidisciplinary teams geared towards the patients, their relatives, and the team members; 10) acceptance of constructive feedback to improve work capacity; and 11) compassion, honesty, and respect for other people.

For these reasons, the technical knowledge of each member per se is not enough in the training of multidisciplinary work teams if there is not a proper understanding of their role in the group. Being multidisciplinary is not enough. The team must to be integrated. Although different levels of those requisites can be found among the members of the group, they can be trained and work in order adjust them to structure the team.

Once the team is formed, it is necessary to establish guidelines (protocols) for delivery rooms, which are essential. The objectives of clinical practice protocols are establishing proper criteria and the algorithm for problem/diagnosis, whether they refer to diseases or bureaucratic events, as well as their treatment or solution, and creating mechanisms to guarantee safe and effective prescriptions and the supervision of possible adverse effects. The documents must to contain technical information based on the best scientific evidence available and obey some primordial principles aiming to improve care quality. They must be clear enough, thus preventing nonconventional procedures and behaviors that have not yet been consolidated, those that are not accessible in Brazil, or those that have not been approved by the Brazilian National Health Surveillance Agency (ANVISA, in Portuguese) for their application. Since most programs are developed in university hospitals, the protocols must be not only the basis for decision-making by the professionals involved, but also an instrument for professional training, excelling through ethical aspects and the preservation of the relationship between professionals and patients.

We recommend, as a model, the recent publication of the ACOG,^10^ which is supported by the American College of Nurse-Midwives (ACOG COMMITTEE OPINION Number 766). After carrying out an extensive review of the international literature, the ACOG Committee has established a series of guidance involving obstetricians, nurses, patients, and all of the professionals that help obstetricians during childbirth. Its main objective, which is common to all of the professionals, is to assist labor and childbirth using techniques that require minimum interventions and result in patient satisfaction.

We believe that all the efforts undertaken by the MH and the other institutions that support programs and projects whose objective is to improve the care offered to pregnant women and parturients will be successful only with permanent investment, mainly regarding infrastructure and the overall development of qualified human resources, with continuing education programs and, most importantly, the integration of all of the professionals involved.
